# Single-Cell RNA-Sequencing From Mouse Incisor Reveals Dental Epithelial Cell-Type Specific Genes

**DOI:** 10.3389/fcell.2020.00841

**Published:** 2020-09-01

**Authors:** Yuta Chiba, Kan Saito, Daniel Martin, Erich T. Boger, Craig Rhodes, Keigo Yoshizaki, Takashi Nakamura, Aya Yamada, Robert J. Morell, Yoshihiko Yamada, Satoshi Fukumoto

**Affiliations:** ^1^Division of Pediatric Dentistry, Department of Oral Health and Development Sciences, Tohoku University Graduate School of Dentistry, Sendai, Japan; ^2^Genomics and Computational Biology Core, National Institute of Dental and Craniofacial Research, National Institutes of Health, Bethesda, MD, United States; ^3^Genomics and Computational Biology Core, National Institute on Deafness and Other Communication Disorders, National Institutes of Health, Bethesda, MD, United States; ^4^Laboratory of Cell and Developmental Biology, National Institute of Dental and Craniofacial Research, National Institutes of Health, Bethesda, MD, United States; ^5^Section of Orthodontics and Dentofacial Orthopedics, Division of Oral Health, Growth, and Development, Kyushu University Faculty of Dental Science, Fukuoka, Japan; ^6^Division of Molecular Pharmacology and Cell Biophysics, Department of Oral Biology, Tohoku University Graduate School of Dentistry, Sendai, Japan; ^7^Division of Oral Health, Growth and Development, Kyushu University Faculty of Dental Science, Fukuoka, Japan

**Keywords:** tooth development, ectodermal organ, gene expression, ameloblast, single-cell RNA-seq

## Abstract

Dental epithelial stem cells give rise to four types of dental epithelial cells: inner enamel epithelium (IEE), outer enamel epithelium (OEE), stratum intermedium (SI), and stellate reticulum (SR). IEE cells further differentiate into enamel-forming ameloblasts, which play distinct roles, and are essential for enamel formation. These are conventionally classified by their shape, although their transcriptome and biological roles are yet to be fully understood. Here, we aimed to use single-cell RNA sequencing to clarify the heterogeneity of dental epithelial cell types. Unbiased clustering of 6,260 single cells from incisors of postnatal day 7 mice classified them into two clusters of ameloblast, IEE/OEE, SI/SR, and two mesenchymal populations. Secretory-stage ameloblasts expressed *Amel* and *Enam* were divided into *Dspp* + and *Ambn* + ameloblasts. Pseudo-time analysis indicated *Dspp* + ameloblasts differentiate into *Ambn* + ameloblasts. Further, *Dspp* and *Ambn* could be stage-specific markers of ameloblasts. Gene ontology analysis of each cluster indicated potent roles of cell types: OEE in the regulation of tooth size and SR in the transport of nutrients. Subsequently, we identified novel dental epithelial cell marker genes, namely *Pttg1*, *Atf3*, *Cldn10*, and *Krt15*. The results not only provided a resource of transcriptome data in dental cells but also contributed to the molecular analyses of enamel formation.

## Introduction

The development processes of ectodermal organs, such as tooth, hair, lung, and kidney, have common features, which are initiated by the interaction of the epithelium with the mesenchyme through the basement membrane (Thesleff, 2003). Tooth development begins from reciprocal interaction of oral epithelium with underneath neural crest-derived mesenchyme. Oral epithelium begins to thicken and becomes dental epithelial stem cells (DESCs). DESCs invaginate into mesenchyme and form tooth germ. During tooth development, DESCs give rise to all types of dental epithelial cells that involve amelogenesis (Juuri et al., 2013). They may be classified into the following five types: ameloblast and its progenitor inner enamel epithelium (IEE), outer enamel epithelium (OEE), cuboidal cells surrounding the outer layer of the enamel organ, stratum intermedium (SI), squamous cells that cover ameloblast, and stellate reticulum (SR), having a spindle shape (Krivanek et al., 2017). All these cell types are essential for proper enamel formation (Liu H. et al., 2016). This classification is based on cell shape, and each cell type seems to play a distinct role; however, the transcriptomic characteristics and roles of OEE and SR are not yet fully understood.

For a better understanding of the molecular mechanism during tooth development, we had previously identified several genes preferentially expressed in ameloblast lineage and had analyzed their roles in tooth development (Saito et al., 2015; Liu J. et al., 2016; Miyazaki et al., 2016; Nakamura et al., 2016; Arai et al., 2017; Han et al., 2018; Nakamura et al., 2020). We had also identified novel genes from tooth germ complementary DNA library, namely *Ameloblastin* (*Ambn*), *Epiprofin* (*Epfn*), and *AmeloD* (Dhamija et al., 1999; Nakamura et al., 2004; He et al., 2019), and each of these knockout mice models showed severe enamel hypoplasia (Fukumoto et al., 2004; Nakamura et al., 2008, 2017; Aurrekoetxea et al., 2016; Chiba et al., 2019). Currently, ameloblast is the best-characterized cell type of dental epithelium and plays the most important role in enamel formation. Enamel formation process, called amelogenesis, can be split into four stages: proliferation stage, secretory stage, transition stage, and maturation stage. The stages are defined by the morphology and function of ameloblasts (Bartlett, 2013). The ameloblasts form a single-cell layer to cover the enamel and play indispensable roles in enamel formation by changing their shape and function through amelogenesis.

DESCs commit cell fate into ameloblast lineages and differentiate into IEE cells, which proliferate and migrate to increase the size of tooth germ during the proliferation stage (Chiba et al., 2019). Soon after, IEE cells exit the cell cycle and become polarized pre-ameloblasts. Pre-ameloblasts extend cytoplasmic projections through the basement membrane to break it. This action enables ameloblasts to deposit the enamel matrix on the dentin–enamel junction (Bartlett, 2013). In the secretory stage, pre-ameloblasts increase in height and differentiate into ameloblasts, which develop Tomes’s processes to secrete enamel matrices such as *Ambn*, *Amelogenin* (*Amel*), and *Enamelin* (*Enam*) (Green et al., 2019). At the end of the secretory stage, ameloblasts lose their Tomes’s processes and produce proteases to degrade and absorb the enamel matrix proteins in the transition stage. In the maturation stage, ameloblasts change their cell shape alternately into a ruffle-ended and smooth-ended phase to modulate ion transportation and pH cycling (Lacruz et al., 2017). Thus, ameloblasts show dynamic changes in their morphology and function to contribute to enamel formation.

Tooth development shows dynamic changes in matrix metabolism and in-cell development, whereas dental epithelium becomes later reduced to enamel epithelium and undergo apoptosis associated with tooth eruption in humans (Lacruz et al., 2017). Therefore, the dental epithelium is a non-regenerative tissue that limits the observation of the developmental trajectory of cell fate. Rodent incisors have stem cell niche in their roots, referred to as a cervical loop, and grow throughout their life (Harada et al., 2006; Sharir et al., 2019).

In the cervical loop region of rodent incisor, *Sox2* + DESCs continuously provide differentiating epithelial cells toward apical sides (Juuri et al., 2013). This model provides a new perspective on the development of dental epithelium. Single-cell RNA sequencing (scRNA-seq) is a powerful tool to clarify the heterogeneity of developing cell types; recent scRNA-seq studies in tooth development have clarified some characteristics of dental epithelial cell types (Landin et al., 2012, 2015; Sharir et al., 2019; Takahashi et al., 2019). However, there has been no report yet that covers the perspective of transcriptome profiles in dental epithelium differentiation, especially amelogenesis using scRNA-seq.

In this study, we performed scRNA-seq analysis using whole mouse incisors to identify the transcriptomic characteristics of enamel-forming dental epithelial cells. The transcriptome map showed the roles of dental epithelial cells and identified potential novel marker genes for each dental cell type. Also, the secretory stage of ameloblasts was categorized as *Dspp* + and *Ambn* + ameloblast clusters, each with a distinct biological role. These findings together demonstrated the establishment of transcriptional identities of dental epithelial cells and uncovered the role of dental epithelial cell types.

## Experimental Procedures

### Animals and Tissues

The Tg(KRT14-RFP)#Efu (Krt14-RFP) mouse line was obtained from Dr. Matthew P. Hoffman and maintained as homozygous (Zhang et al., 2011). The animal protocol used in the present study was approved by the National Institute of Dental and Craniofacial Research (NIDCR) Animal Care and Use Committee (protocol number ASP16-796). All animals were housed in a facility approved by the American Association for the Accreditation of Laboratory Animal Care. Incisors were dissected with sharp tweezers from seven littermates of P7 Krt14-RFP mice. Single-cell dissociation was essentially performed as previously described (Li et al., 2015). Briefly, dissected incisors were incubated in 4-mg/ml Dispase II (Roche) for 14 min at 37°C. They were placed in cold Dulbecco’s modified Eagle medium/F12, and dental epithelium and mesenchyme were separated under a microscope. The tissues are then placed in Accutase (Sigma) for 30 min at 37°C. After pipetting up and down, using 1,000 μm tips, a single-cell suspension was produced by passing through a 70 μm sterile cell strainer. The cells were resuspended with 0.04% bovine serum albumin containing cold phosphate-buffered saline. All methods were carried out following relevant guidelines and regulations.

### Single-Cell Library Preparation and Sequencing

Single-cell library preparation was performed following the manufacturer’s instructions for the 10 × Chromium single-cell kit (10x Genomics, CA, United States). The libraries were sequenced on a NextSeq 500 sequencer (Illumina, CA, United States), as previously described (Sekiguchi et al., 2020).

### Single-Cell RNA Sequencing Data Processing and Quality Control

Read processing was performed using the 10 × Genomics workflow (Zheng et al., 2017). Briefly, the Cell Ranger Single-Cell Software Suite was used for demultiplexing, barcode assignment, and unique molecular identifier (UMI) quantification^1^. The reads were aligned to the mm10 reference genome (Genome Reference Consortium Mouse Build 38) using a pre-built annotation package obtained from the 10 × Genomics website^2^. Samples were demultiplexed using the “cellranger mkfastq” function, and gene count matrices were generated using the “cellranger count” function.

The Cell Ranger software identified, on an average, 46,146 barcodes per sample, containing 7,356 median UMIs per cell. The mean sequencing saturation of the sample was 50%. The following metrics were used to flag poor-quality cells: number of genes detected, total number of UMIs, and percentage of molecules mapped to mitochondrial genes. Data for specific cells were not included in subsequent analyses when fewer than 700 genes were detected. Cells, with a mean of >9% of UMIs mapped to mitochondrial genes, were defined as non-viable or apoptotic and were excluded from the analyses. Genes expressed in <5 cells were not included; 6,260 cells were included in subsequent clustering.

### Cell Clustering Analyses

Secondary analysis and filtering were performed using the Seurat v2 R package (Satija et al., 2015). To assign epithelial and mesenchymal cells to distinct clusters based on differentially expressed transcripts, significant dimensions were first defined by principal component analysis. The number of significant PCs for clustering analysis was determined by the “JackStraw” function implemented by the Seurat package at *p* < 0.05 with 15 PCs. The significant PCs were applied to graph-based clustering using Seurat’s “findClusters” function. Cluster representations were performed by t-distributed stochastic neighbor embedding (t-SNE) at resolution 0.2.

### Differential Expression, Gene Ontology, and Pseudo-Time Analysis

Per cluster differential gene expression was computed with Seurat2’s “FindAllMarkers” function using default parameters (Zheng et al., 2017). The Log_2_FoldChange was the ratio of gene expression of one cluster to that of all other cells. The *P*-value was calculated by a negative binomial exact test (Yu et al., 2013). We considered marker genes that were differentially expressed (*p* < 0.01). For gene ontology (GO) analysis, an online platform for GO enrichment analysis, provided by the Gene Ontology Consortium^3^, was used (Ashburner et al., 2000; Mi et al., 2019) with the differentially expressed in each cluster (*p* < 0.01). The R package monocle was used to perform pseudo-time analysis. To cluster genes by pseudo-temporal expression pattern, the function “plot_pseudotime_heatmap” was used (Trapnell et al., 2014).

### Immunofluorescence and Single-Molecule Fluorescence *in situ* Hybridization

P1 and P3 mouse heads were embedded in paraffin, sectioned, and subjected to immunofluorescence, as described previously (Chiba et al., 2019). The primary antibodies of AMEL (Abcam, 1:200), NOTCH2 (Cell Signaling Technology, 1:200), PTTG1 (Abcam, 1:100), CLDN10 (Thermo Fisher Scientific, 1:200), ATF3 (Abcam, 1:100), and KRT15 (Abcam, 1:200) were used for immunostaining. These primary antibodies were detected by Alexa Fluor 488-conjugated antibody (Invitrogen, 1:400). Nuclear staining was performed with DAPI (Sigma).

For single-molecule fluorescence *in situ* hybridization (smFISH), Custom Stellaris^®^ Probe Sets (LGC Biosearch Technology, Hoddesdon, United Kingdom) for mouse Ambn and mouse Dspp were designed by Stellaris Probe Designer. Hybridization buffer was prepared as previously described (Wang, 2019), and smFISH was performed following the manufacturer’s protocol.

## Results

### Single-Cell Transcriptome Analysis of Mouse Incisors Showed the Entire Population of Dental Cells

**FIGURE 1:**
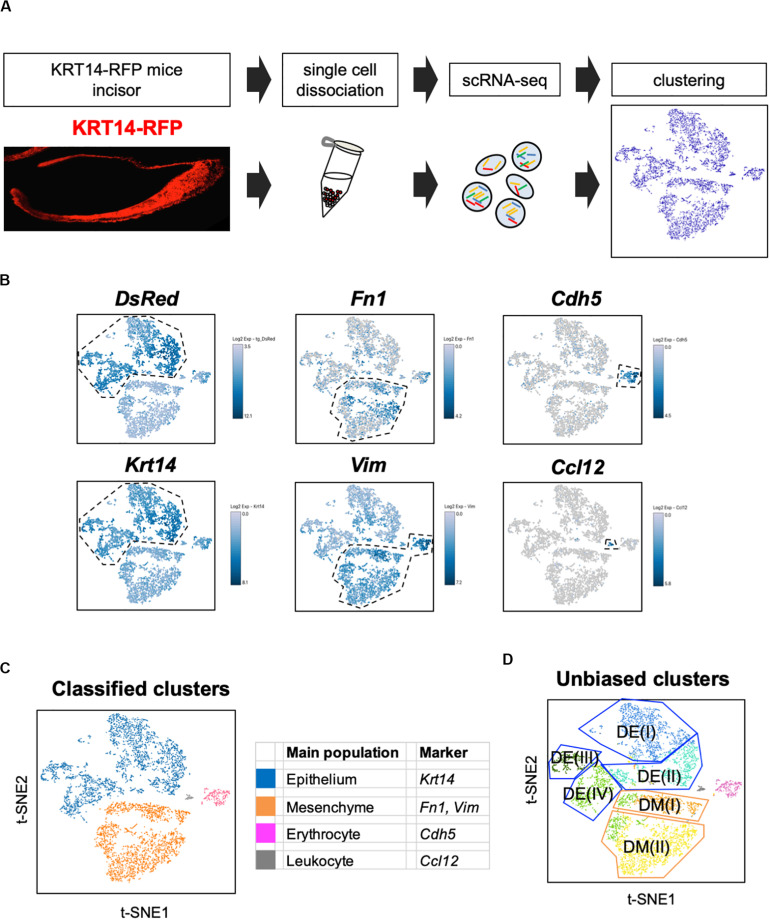
t-SNE visualization showed the entire population of the incisor. **(A)** Schematic representation of the procedure for single-cell RNA-sequencing using Krt14-RFP P7 mouse incisors. Incisors were dissected from postnatal day 7 (P7) Krt14-RFP littermates. Left panel shows a sagittal section of P3 Krt14-RFP mouse incisor. **(B)** Expression of cell-type-specific marker genes projected onto the t-SNE plot. *DsRed* and *Krt14*: epithelium, *Fn1* and *Vim*: mesenchyme, *Cdh5*: endothelial cell, *Ccl12*: leukocyte. **(C)** Classified population based on the marker genes analyzed in Figure 1B. The four clusters comprised of *Krt14* + epithelium (blue), *Fn1* + mesenchyme (orange), *Cdh5* + endothelial cell (pink), and *Ccl12* + leukocyte (gray). **(D)** t-SNE visualization of all single-cell RNA-seq datasets from P7 Krt14-RFP mouse incisors. Automatic clustering based on log-transformed mean expression values of the eight clusters, identifying four epithelial, two mesenchymal, erythrocyte, and leukocyte clusters. Blue lines indicate epithelial clusters. Orange lines indicate mesenchymal clusters. DE, dental epithelium; DM, dental mesenchyme.

We first established a fluorescent mouse model, to distinguish dental epithelium from other cell types, using Tg(KRT14-RFP)#Efu, a *Keratin14* (*Krt14*)-promoter driven RFP tag mouse (Krt14-RFP) (Zhang et al., 2011). *Krt14* is a marker of the dental epithelium (Chavez et al., 2014), and in Krt14-RFP mice, RFP was observed in the entire dental epithelium in postnatal-day (P) 3 incisors (Figure 1A). Rodent incisor develops continuously throughout their life; therefore, it is a good model to analyze the developing cell stages. Although maturation of mice incisors takes 4 weeks after their birth, most of the cells that consist of incisor are observed at P7. To isolate all types and stages of dental cells, we dissected mandibular incisors of P7 Krt14-RFP mice littermates and dissociated dental cells into single cells. Single cells were immediately loaded to the 10 × genomics platform, and scRNA-seq was performed. We obtained 6,260 single-cell transcriptome data with 2,386 mean genes per cell (Table 1). Differential gene expression analysis identified *DsRed* + (*Krt14* +) epithelial cell, *Fn1* + mesenchymal cell, *Cdh5* + endothelial cell, and *Ccl12* + leukocyte clusters (Figures 1B,C).

**TABLE 1:** Quality control statistics for scRNA-seq.

	P7 incisor
Number of cells captured	6,260
Mean reads per cell	46,146
Mean genes per cell	2,386
Sequencing saturation (%)	50

### Classification Based on Marker Genes Showed the Known Dental Cell Populations and Their Transcriptomic Characteristics

**FIGURE 2:**
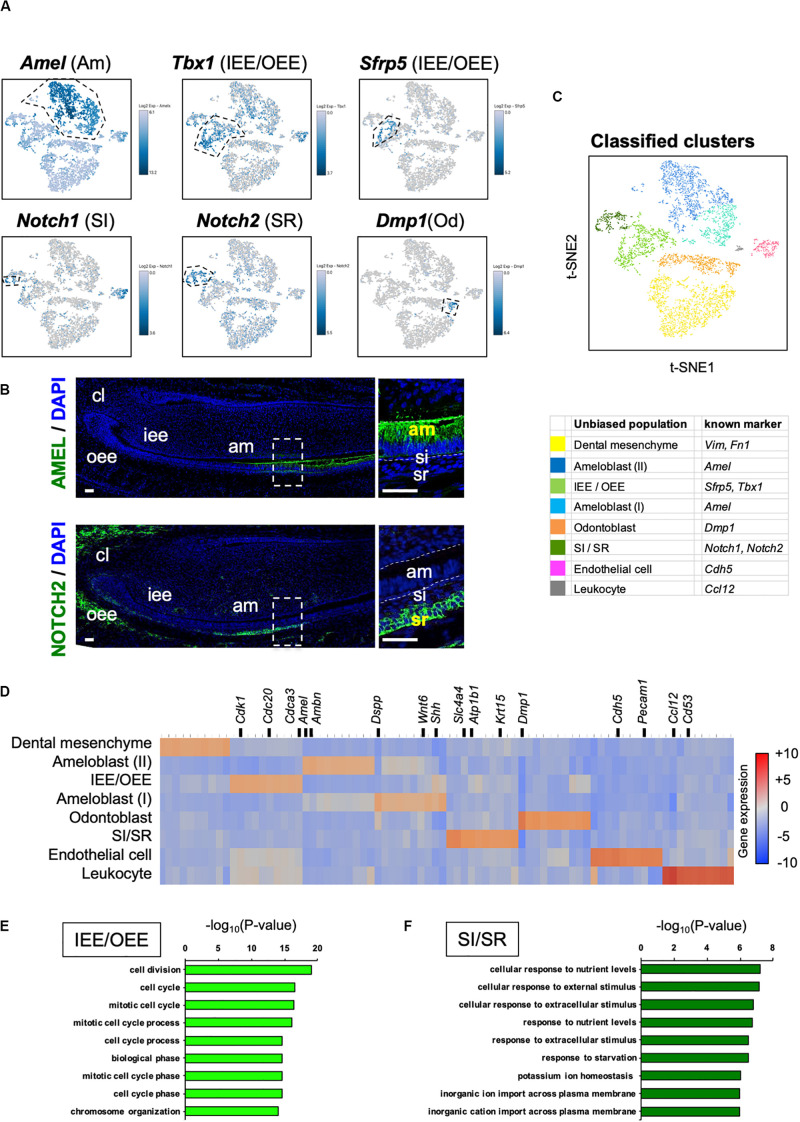
Unbiased clusters were profiled by cell type marker genes. **(A)** Expression of known dental cell-type-specific marker genes projected onto the t-SNE plot. *Amel*: ameloblast, *Tbx1*: IEE and OEE, *Sfrp5*: IEE and OEE, *Notch1*: SI, *Notch2*: SR, *Dmp1*: odontoblast (Od). **(B)** Immunofluorescence of AMEL, NOTCH2 in P1 mouse incisors. AMEL localized in ameloblast. NOTCH2 localized in SR. Boxed area was magnified in the right panel. AM, ameloblast; CL, cervical loop; IEE, inner enamel epithelium; OEE; outer enamel epithelium; SI, stratum intermedium; SR, stellate reticulum. Scale bars: 50 μm. **(C)** Classified population based on the dental cell-type marker genes analyzed in Figure 2A. Unbiased eight clusters included two clusters of Amel + ameloblast (blue and light blue), *Tbx1* + and *Sfrp5* + IEE/OEE cluster (light green), *Notch1* + and *Notch2* + SI/SR cluster (green), *Dmp1* + odontoblast (orange), *Fn1* + and *Vim* + mesenchyme (yellow), *Cdh5* + endothelial cell (pink), and *Ccl12* + leukocyte (gray). **(D)** Heatmap analysis demonstrated 50 highly differentially expressed genes between clusters. Genes known as cell type markers are labeled. Bar plots highlight GO terms enriched in the cluster of **(E)** IEE/OEE and **(F)** SI/SR with differentially expressed genes.

Unbiased clustering identified four epithelial and two mesenchymal clusters, excluding erythrocyte and leukocyte clusters (Figure 1D). We first analyzed the expression of dental cell marker genes to identify the known dental cell populations in the t-SNE plot. *Amel*, *Tbx1*, *Sfrp5*, *Notch1*, and *Notch2* were used as cell type-specific marker genes, as reported previously (Harada et al., 1999; Mitsiadis et al., 2010; Juuri et al., 2013). The secretory stage of ameloblasts was marked by Amel, and IEE and OEE were characterized by *Tbx1* and *Sfrp5* (Figure 2A). SI cell appeared as a high *Notch1*-expressing cluster in the epithelium, whereas SR cell showed a high expression of *Notch2*. *Dmp1* marked odontoblast in mesenchymal clusters. We confirmed the expression pattern of AMEL and NOTCH2 by immunofluorescence in the P1 mouse incisor (Figure 2B). As expected, AMEL and NOTCH2 were localized in ameloblast and SR cell, respectively. Based on marker gene expression, the dental epithelial population was classified into two epithelial clusters of the *Amel* + secretory stage of ameloblast Ameloblasts (I) and (II), a *Tbx1* + /*Sfrp5* + epithelial cluster IEE/OEE, and *Notch1* + /*Notch2* + epithelial cluster SI/SR (Figure 2C). Interestingly, unbiased clustering indicated IEE and OEE, or SI and SR cells, to have similarity in the transcriptome, and categorized the cell types into one cluster. Although IEE/OEE or SI/SR clusters were separate, they were located closely in t-SNE visualization. We further examined the expression of developmental stage-dependent genes in the t-SNE plot (Supplementary Figure 1). Ki67 (*Mki67*) is a marker gene for actively proliferating cells, which was observed in the IEE/OEE cluster. *Shh* and *Wnt6* are known to be expressed in enamel knot and pre-ameloblast (Gritli-Linde et al., 2002; Cobourne et al., 2004; Wang et al., 2010), which was observed in IEE/OEE clusters and Ameloblast clusters, with especially high expression in Ameloblast (I) cluster. These results indicate the differentiation processes of ameloblast lineages.

We next examined the transcriptomic characteristics of each cluster by differential gene expression analysis (Figure 2D). Heatmap showed obvious differences in the highly expressed genes across cell clusters, suggesting them to have distinct roles in tooth development. Notably, IEE/OEE cells highly expressed cell cycle-related genes, such as *Cdk1*, *Cdc20*, and *Cdca3* (Figure 2E). Although *Notch1* and *Notch2* were specifically expressed in SI and SR cells, respectively, they were not included in the top 20 highly expressed genes (Table 2 and Supplementary Figure 2). GO analyses were performed with the highly expressed genes in IEE/OEE and SI/SR clusters to clarify the roles of each cell type during tooth development (Figures 2E,F). In IEE/OEE clusters, GO terms were mostly enriched in cell division and cell cycle, in agreement with their high proliferation activity (Figure 2E). In SI/SR clusters, GO enrichment indicated that they act as regulators of nutrient and ion transport (Figure 2F), in line with previous reports (Kallenbach, 1978; Inage et al., 1987; Yoshida et al., 1989; Nakamura and Ozawa, 1997).

**TABLE 2:** Top 20 highly expressed genes of each cluster.

Gene	Cluster	*p*-value	Log FC
*Dspp*	Ameloblast (I)	4.59E-21	2.7
*Cyp26a1*	Ameloblast (I)	5.08E-14	2.3
*Col9a3*	Ameloblast (I)	2.01E-13	2.2
*Stac3*	Ameloblast (I)	3.21E-11	2.0
*Acp5*	Ameloblast (I)	1.99E-04	1.9
*Fxyd4*	Ameloblast (I)	2.45E-10	1.9
*Plod2*	Ameloblast (I)	1.22E-09	1.8
*Calb1*	Ameloblast (I)	2.12E-09	1.8
*H2-Eb1*	Ameloblast (I)	2.95E-02	1.8
*Fxyd3*	Ameloblast (I)	5.52E-09	1.8
*Wnt6*	Ameloblast (I)	7.98E-08	1.7
*Ndrg1*	Ameloblast (I)	4.78E-07	1.6
*Nrn1l*	Ameloblast (I)	2.56E-06	1.6
*tg_DsRed*	Ameloblast (I)	2.89E-06	1.5
*Acpp*	Ameloblast (I)	6.39E-06	1.5
*Cd24a*	Ameloblast (I)	4.57E-06	1.5
*Shh*	Ameloblast (I)	2.15E-05	1.5
*Krt14*	Ameloblast (I)	6.61E-06	1.5
*Krt5*	Ameloblast (I)	3.03E-05	1.4
*Ambn*	Ameloblast (II)	1.71E-26	2.5
*Amelx*	Ameloblast (II)	8.15E-25	2.5
*Chst1*	Ameloblast (II)	5.36E-18	2.1
*Acpt*	Ameloblast (II)	3.64E-17	2.1
*Lama3*	Ameloblast (II)	1.39E-16	2.1
*Aplp1*	Ameloblast (II)	9.29E-17	2.0
*Cd55*	Ameloblast (II)	5.48E-15	2.0
*Galnt12*	Ameloblast (II)	2.95E-14	1.9
*Lamb3*	Ameloblast (II)	6.66E-14	1.9
*Enam*	Ameloblast (II)	2.76E-13	1.8
*Arsb*	Ameloblast (II)	1.57E-11	1.7
*Cntnap2*	Ameloblast (II)	1.57E-11	1.7
*Itpr1*	Ameloblast (II)	1.89E-10	1.6
*Hba-a2*	Ameloblast (II)	2.77E-06	1.6
*Cystm1*	Ameloblast (II)	1.26E-09	1.6
*Fam46a*	Ameloblast (II)	3.00E-09	1.5
*Itpripl2*	Ameloblast (II)	4.50E-08	1.4
*Lamc2*	Ameloblast (II)	2.68E-07	1.4
*Fam3c*	Ameloblast (II)	3.35E-07	1.4
*Epcam*	IEE/OEE	6.17E-13	2.1
*Mt2*	IEE/OEE	2.93E-12	2.1
*Pitx2*	IEE/OEE	1.88E-09	1.8
*Shh*	IEE/OEE	4.49E-09	1.8
*Pdgfa*	IEE/OEE	1.86E-08	1.7
*Ube2c*	IEE/OEE	5.90E-08	1.7
*Ccnd1*	IEE/OEE	1.05E-07	1.7
*Hmgb2*	IEE/OEE	6.19E-07	1.6
*Cenpf*	IEE/OEE	2.51E-06	1.6
*Cenpa*	IEE/OEE	2.01E-06	1.6
*Sostdc1*	IEE/OEE	1.53E-06	1.5
*Stmn1*	IEE/OEE	1.39E-06	1.5
*Lgals7*	IEE/OEE	3.49E-06	1.5
*Cyp26a1*	IEE/OEE	5.96E-06	1.5
*2810417H13Rik*	IEE/OEE	4.98E-06	1.5
*Pfdn4*	IEE/OEE	4.51E-06	1.5
*Krt17*	IEE/OEE	1.18E-05	1.4
*Cldn10*	IEE/OEE	6.64E-05	1.4
*Smc4*	IEE/OEE	1.64E-05	1.4
*Tfrc*	SI/SR	1.06E-40	4.6
*Chchd10*	SI/SR	1.53E-27	3.8
*Slc4a4*	SI/SR	4.50E-27	3.9
*Atp1b1*	SI/SR	1.58E-25	3.9
*Nectin3*	SI/SR	1.29E-24	3.7
*Igfbp2*	SI/SR	8.53E-23	3.6
*Krt15*	SI/SR	6.69E-21	3.5
*Gnrh1*	SI/SR	7.28E-20	3.5
*Sgk1*	SI/SR	1.84E-19	3.4
*Dapl1*	SI/SR	1.87E-19	3.5
*Dsc3*	SI/SR	3.65E-19	3.4
*Barx2*	SI/SR	8.93E-19	3.3
*Snap91*	SI/SR	9.99E-19	3.3
*Tnc*	SI/SR	1.44E-18	3.4
*Atp1b3*	SI/SR	1.77E-17	3.2
*Tst*	SI/SR	6.21E-16	3.1
*Rbbp8*	SI/SR	1.90E-14	3.0
*Fam13a*	SI/SR	1.39E-13	2.9
*Dsp*	SI/SR	1.42E-13	2.9
*Dmp1*	Odontoblast	1.12E-27	4.9
*Smpd3*	Odontoblast	1.97E-50	4.5
*Plac8*	Odontoblast	9.56E-40	4.0
*Bglap*	Odontoblast	1.63E-20	3.9
*Sct*	Odontoblast	4.33E-24	3.9
*Bglap2*	Odontoblast	1.00E-17	3.9
*Dkk1*	Odontoblast	1.61E-28	3.8
*Bglap3*	Odontoblast	7.34E-18	3.7
*Sgms2*	Odontoblast	1.69E-29	3.6
*Ifitm5*	Odontoblast	1.32E-24	3.4
*Dcn*	Odontoblast	4.01E-15	2.6
*Cox4i2*	Odontoblast	2.05E-13	2.5
*Gchfr*	Odontoblast	4.74E-12	2.4
*Omd*	Odontoblast	2.12E-11	2.3
*Ibsp*	Odontoblast	3.60E-06	2.3
*Lum*	Odontoblast	6.64E-12	2.3
*Ptn*	Odontoblast	1.09E-11	2.3
*Serpinf1*	Odontoblast	1.10E-10	2.2
*Cdkn1c*	Odontoblast	1.24E-08	2.0
*Mpz*	Mesenchyme	3.67E-06	2.8
*Ogn*	Mesenchyme	1.13E-28	2.8
*Sfrp2*	Mesenchyme	4.95E-23	2.7
*Mmp13*	Mesenchyme	5.13E-23	2.7
*Spp1*	Mesenchyme	1.24E-11	2.7
*Bmp3*	Mesenchyme	1.13E-24	2.6
*Sfrp4*	Mesenchyme	4.93E-23	2.6
*Tagln*	Mesenchyme	5.72E-24	2.6
*Col12a1*	Mesenchyme	1.62E-22	2.5
*Ncam1*	Mesenchyme	2.26E-22	2.5
*Postn*	Mesenchyme	4.58E-20	2.4
*Col3a1*	Mesenchyme	9.13E-23	2.4
*Igf1*	Mesenchyme	2.17E-22	2.4
*Mbp*	Mesenchyme	1.24E-05	2.4
*Nts*	Mesenchyme	4.03E-21	2.4
*Col1a2*	Mesenchyme	2.15E-20	2.3
*Sfrp1*	Mesenchyme	1.36E-18	2.2
*Acta2*	Mesenchyme	1.73E-17	2.2
*Alx1*	Mesenchyme	1.73E-18	2.2
*Ccl12*	Leukocyte	1.64E-09	8.0
*Cd209f*	Leukocyte	5.12E-06	7.5
*Cd209g*	Leukocyte	1.36E-04	7.3
*Mrc1*	Leukocyte	2.15E-15	7.1
*Clec10a*	Leukocyte	8.61E-12	7.1
*Cd86*	Leukocyte	5.55E-14	6.9
*Ms4a6c*	Leukocyte	5.11E-20	6.8
*Ly86*	Leukocyte	1.11E-13	6.8
*C1qc*	Leukocyte	9.38E-21	6.7
*Adgre1*	Leukocyte	8.73E-15	6.7
*Ms4a6d*	Leukocyte	1.20E-15	6.7
*Ccl4*	Leukocyte	6.94E-10	6.7
*Ccl3*	Leukocyte	3.24E-12	6.6
*Cx3cr1*	Leukocyte	3.57E-17	6.6
*Ms4a7*	Leukocyte	3.20E-18	6.6
*Fcrls*	Leukocyte	6.42E-15	6.6
*Ms4a6b*	Leukocyte	2.86E-15	6.5
*Fcgr3*	Leukocyte	2.49E-16	6.5
*Clec4n*	Leukocyte	6.42E-15	6.4
*Cdh5*	Endothelial cell	2.54E-58	5.2
*Lmo2*	Endothelial cell	3.94E-45	5.1
*Ccl21a*	Endothelial cell	2.01E-10	5.1
*Flt1*	Endothelial cell	3.58E-51	5.0
*Pecam1*	Endothelial cell	1.93E-48	4.8
*Slco2a1*	Endothelial cell	2.02E-38	4.8
*Myct1*	Endothelial cell	1.51E-44	4.7
*Clec14a*	Endothelial cell	2.91E-43	4.7
*Kdr*	Endothelial cell	3.94E-45	4.7
*Plvap*	Endothelial cell	3.21E-43	4.6
*Cd34*	Endothelial cell	2.94E-44	4.6
*Adgrf5*	Endothelial cell	5.11E-41	4.5
*Cd93*	Endothelial cell	1.52E-42	4.5
*Fabp4*	Endothelial cell	1.15E-24	4.5
*Apold1*	Endothelial cell	4.24E-40	4.4
*Aplnr*	Endothelial cell	3.11E-38	4.4
*Emcn*	Endothelial cell	2.05E-40	4.4
*Cldn5*	Endothelial cell	2.93E-37	4.4
*Rgcc*	Endothelial cell	1.26E-37	4.3

### Differential Gene Expression Analysis Identified Novel Marker Genes in Dental Epithelial Cells

**FIGURE 3:**
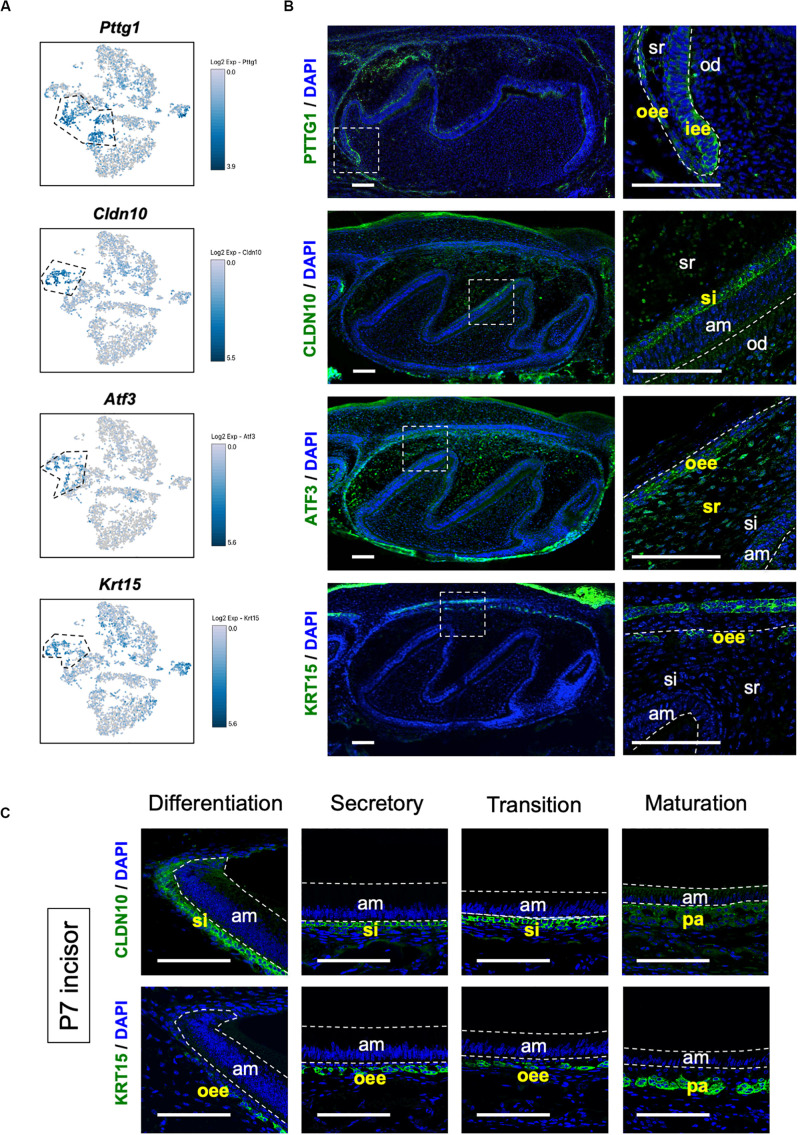
Novel marker genes of dental epithelial cell types were identified by differential expression analysis. **(A)** Expression of novel dental cell-type-specific marker genes projected onto the t-SNE plot. *Pttg1*, *Cldn10*, *Atf3*, and *Krt15* were identified as candidate marker genes from differentially expressed genes in each cluster. **(B)** Immunofluorescence of novel dental cell-type-specific marker genes in P1 mouse molars. PTTG1 localized in IEE and OEE. CLDN10 localized in SI. ATF3 localized in OEE and SR. KRT15 localized in OEE. Boxed area was magnified in the right panel. AM, ameloblast; IEE, inner enamel epithelium; OD, odontoblast; OEE; outer enamel epithelium; SI, stratum intermedium; SR, stellate reticulum. Scale bars: 100 μm. **(C)** Immunofluorescence of CLDN10 and KRT15 in P7 mouse incisors. CLDN10 was continuously localized in SI, although the papillary layer showed weak expression. KRT15 localized in the outer enamel epithelium and outer layer of the papillary layer. AM, ameloblast; OEE; outer enamel epithelium; SI, stratum intermedium; PA, papillary layer. Scale bars: 100 μm.

We sought to identify novel marker genes in dental epithelial cells. We selected several genes that were specifically expressed in IEE/OEE or SI/SR clusters (Figure 3A). Immunofluorescence was performed in P1 mouse molars to examine the localization of *Pituitary tumor-transforming 1* (*Pttg1*), *Claudin 10* (*Cldn10*), *Activating transcription factor 3* (*Atf3*), and *Keratin 15* (*Krt15*) (Figure 3B). At this stage, molars have distinct differentiated cell types. PTTG1 was localized in OEE and IEE cells. CLDN10 was expressed in SI cells. ATF3 was expressed in both OEE and SR cells. KRT15 was remarkable in OEE. We further examined the expression of CLDN10 and KRT15 in P7 incisors (Figure 3C). As expected, CLDN10 was localized in SI cells and KRT15 was expressed in OEE cells. These results together indicated the genes to be novel dental epithelial cell markers.

### Unbiased Clustering Revealed Novel Subpopulations of Ameloblast

**FIGURE 4:**
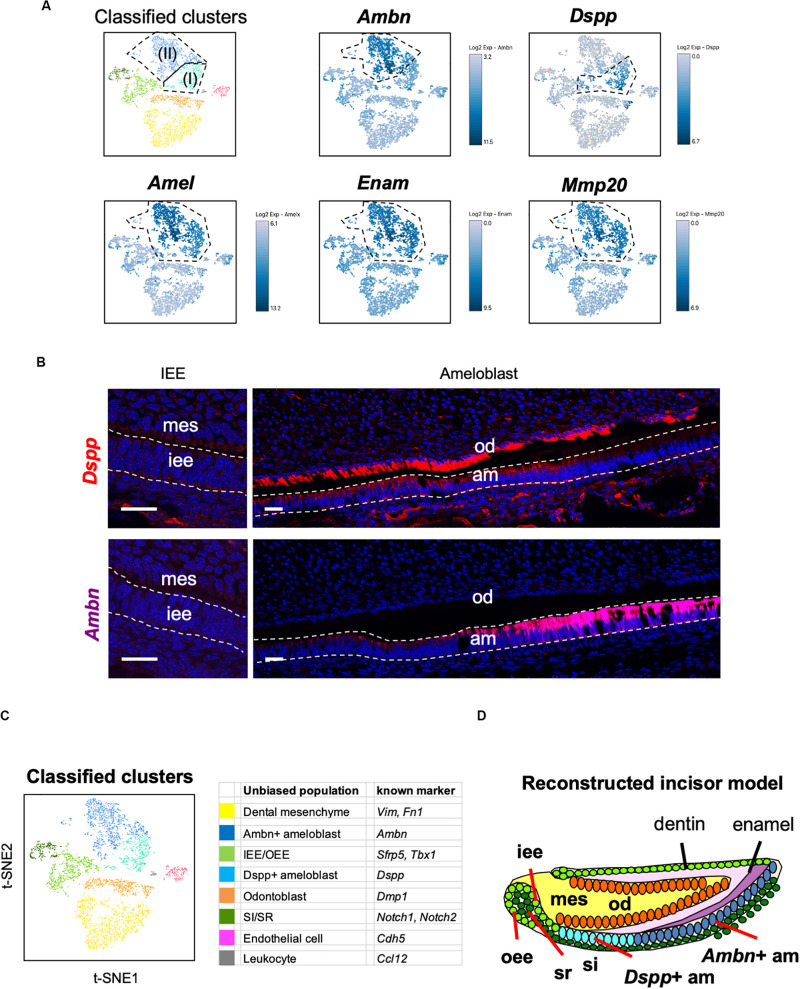
Main population of ameloblasts was divided into Dspp-positive and Ambn-positive clusters. **(A)** Expression of ameloblast marker genes projected onto the t-SNE plot. Secretory-stage ameloblast marker genes (*Amel*, *Enam*, and *Mmp20*) were expressed in the entire ameloblast clusters, whereas *Ambn* + and *Dspp* + clusters were separated by the unbiased population. **(B)** Single-molecule fluorescence *in situ* hybridization (smFISH) of Ambn and Dspp in P1 mouse incisor. Left panel, IEE region. Right panel, ameloblast region. AM, ameloblast; IEE, inner enamel epithelium; MES, dental mesenchyme; OD, odontoblast. Scale bars: 100 μm. **(C)** Classified populations based on the marker genes of dental epithelial cell types analyzed in **(A)**. Secretory-stage ameloblast clusters were divided into *Ambn* + ameloblast and *Dspp* + ameloblast. **(D)** Model of reconstructed incisors based on the classification in **(C**). AM, ameloblast; IEE, inner enamel epithelium; MES, dental mesenchyme, OD; odontoblast, OEE; outer enamel epithelium; SI, stratum intermedium; SR, stellate reticulum.

Unbiased clustering showed that the *Amel* + secretory stage of ameloblasts could be divided into two subpopulations, namely Ameloblasts (I) and (II), as shown in Figure 2C. To characterize these clusters, we first analyzed the expression of secretory-stage ameloblast marker genes in scRNA-seq datasets. *Ambn*, *Amel*, *Enam*, and *Matrix metalloproteinase 20* (*Mmp20*) are well-investigated secretory-stage ameloblast markers (Fukumoto et al., 2004; Bartlett, 2013). *Enam* and *Mmp20* were seen in both ameloblast clusters, similar to the expression of *Amel*; however, *Ambn* was highly expressed in Ameloblast (II) (Figure 4A). In contrast, *Dspp* was specifically expressed in the Ameloblast (I) cluster. We next examined the localization of *Ambn* and *Dspp* messenger RNAs in P1 mouse incisors by smFISH (Figure 4B). Although *Dspp* was expressed in differentiated odontoblasts and early-stage secretory ameloblasts, later on, it was not found to be expressed in fully differentiated ameloblasts. *Ambn* was found to gradually increase its expression from early-stage differentiation and showed high expression in fully-differentiated ameloblasts. These results clarified that Ameloblast (II) cluster contained *Ambn* + fully differentiated ameloblasts, and Ameloblast (I) cluster contained *Dspp* + early-differentiated ameloblast subpopulation (Figures 4C,D).

### Transcriptomic Characterization Indicated Distinct Roles of *Dspp* + Ameloblast and *Ambn* + Ameloblast

DESCs differentiate into IEE cells at the proliferative stage and into pre-ameloblasts after that. The secretion of enamel matrices by ameloblasts with *Amel*, *Enam*, and *Mmp-20* expression defines the secretory stage of ameloblasts (Bartlett, 2013). Currently, the secretory stage of ameloblasts has been defined as consisting of one population. Our current results indicated that ameloblasts actually have two distinct subpopulations (Figures 4A,C). Moreover, *Dspp* and *Ambn* could be spatiotemporal marker genes of these stages.

**FIGURE 5:**
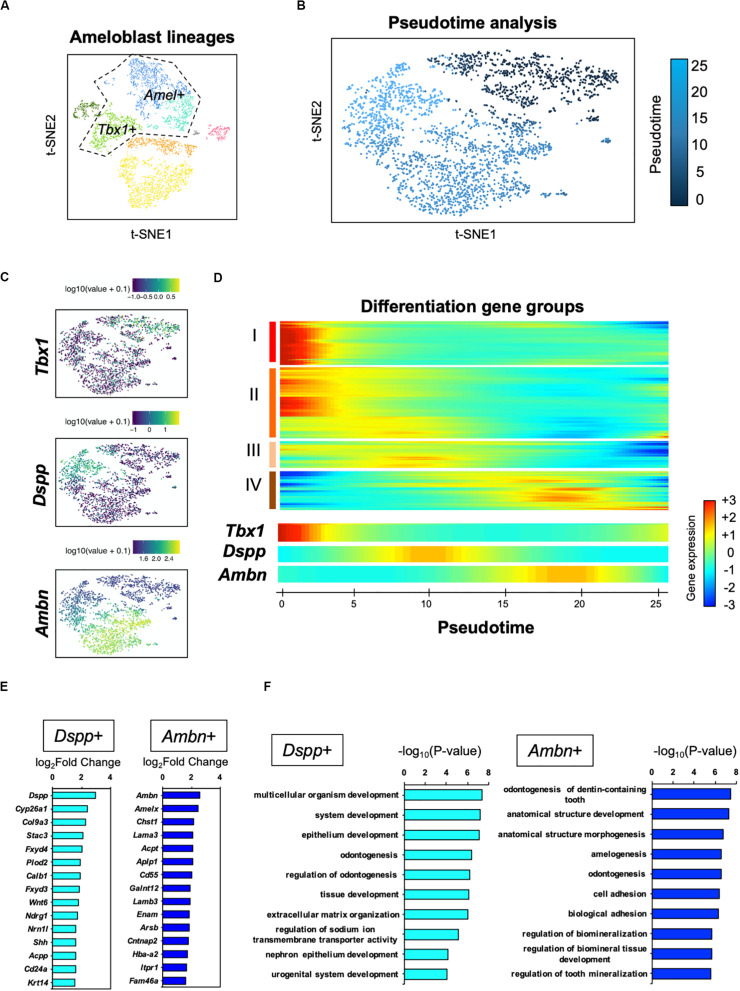
Pseudo-time analysis indicated the process of ameloblast development. **(A)** Ameloblast lineages were used for the pseudo-time analysis as input. Dotted line indicates input dataset. **(B)** Pseudo-temporal ordering of ameloblast lineages (*n* = 2,184) in t-SNE plot. **(C)** Expression of ameloblast marker genes projected onto the t-SNE plot in Figure 5A. *Tbx1* is used as an IEE marker. **(D)** Rolling wave analysis demonstrated the spline-smoothed expression pattern of pseudo-time-dependent genes. Differentiation gene groups clustered into four groups (I–IV) according to their peak expression. **(E)** Fifteen highly expressed genes in the *Dspp* + cluster (left) and *Ambn* + cluster (right) compared with those in the others. **(F)** Bar plots highlight GO terms enriched in the clusters of *Dspp* + (left) and *Ambn* + (right) with differentially expressed genes.

For further analysis of the ameloblast developmental trajectory, we performed pseudo-time analysis using an algorithm of Monocle 2 (Trapnell et al., 2014). First, we tested the entire differentiation process of whole dental epithelial cells by trajectory analysis (Supplementary Figure 3). The datasets of whole dental epithelial cells were used as input (Supplementary Figure 3A) and pseudo-temporal plots were represented in trajectory analysis (Supplementary Figure 3B). Differential expression analysis of cell-type-specific genes, which was used in Figure 2A, revealed that the trajectory was divided into IEE/OEE, SI/SR, and Ameloblast branches (Supplementary Figure 3C). With the results of pseudo-temporal plots, trajectory analysis indicated that IEE/OEE clusters gave rise to SI/SR and Ameloblast clusters. We then further analyzed the detailed differential trajectory in ameloblast lineages (Figure 5). *Tbx1* + IEE/OEE and *Amel* + ameloblast clusters were used as input datasets of ameloblast lineages (Figure 5A). Pseudo-temporal plots were represented in t-SNE plots, showing the trajectory of ameloblast differentiation (Figure 5B). *Tbx1*, *Dspp*, and *Ambn* were expressed in different clusters of pseudo-temporal t-SNE visualization (Figure 5C). We next tested the pseudo-time-dependent clustering of gene expression. In the “Rolling wave” plot, the genes showing a significant change in expression, based on pseudo-time axis, were picked up as differentiation genes. Differentiation gene groups were clustered into four groups: (I) to (IV) (Figure 5D). *Tbx1* showed a similar pattern with the groups (I) and (II), whereas *Dspp* was seen in group (III), and *Ambn* showed similarity with group (IV). These results indicate that the *Dspp* + cluster has a distinct state and later differentiates into *the Ambn* + cluster. This corresponds to the finding obtained from smFISH in Figure 4B. We checked the highly expressed genes in the *Dspp* + and *Ambn* + clusters (Figure 5E). Signaling molecules, such as *Wnt6* and *Shh*, were highly expressed in *Dspp* + cells, thus indicating that they promoted further differentiation into *Ambn* + cells. In *Ambn* + clusters, extracellular matrix genes, including those of enamel matrices (*Ambn*, *Amelx*, *Lama3*, *Lamb3*, and *Enam*), were highly expressed, suggesting their potential to produce enamel matrices. GO analysis of the *Dspp* + cluster showed enriched term in an epithelial organization, whereas the *Ambn* + cluster was enriched in the term indicating enamel formation, such as amelogenesis, adhesion, and mineralization (Figure 5F). These results together suggested ameloblasts as having two distinct cell subpopulations, namely *Dspp* + ameloblast and *Ambn* + ameloblast, at the transcriptome level.

## Discussion

Our transcriptome data showed the entire population in P7 mouse incisor and identified novel subpopulation of secretory-stage ameloblasts, seen as *Dspp* + early-differentiated ameloblast and *Ambn* + fully differentiated ameloblast. P7 incisor has DESCs in the cervical loop region and fully differentiated cells in the apical region (Jheon et al., 2011). We could observe all the stages and types of developing dental epithelium in scRNA-seq datasets. Transcriptome analysis clearly divided the dental epithelial cell population into four clusters, *Ambn* + ameloblast (II), *Dspp* + ameloblast (I), IEE/OEE, and SI/SR clusters (Figures 2C, 4C). Results indicated IEE and OEE or SI and SR to possibly have common genes and roles during tooth development to some extent. This finding agreed with the previous single-cell analysis of the cervical loop in incisors (Sharir et al., 2019). Further, we identified *Pttg1*, *Cldn10*, *Atf3*, and *Krt15* as novel markers of dental epithelial cell type (Figure 3). Interestingly, *Pttg1* localized in both IEE and OEE; it may be one of the candidate marker genes of the IEE/OEE cluster. Also, we found the population of the transition and maturation stages of ameloblasts to be composed of *Amtn* + /*Klk4* + cells or *Slc24a4* + cells, respectively (Supplementary Figure 4), however, they were not categorized as the main population in the current dataset owing to the limitation in captured cell number. Further analysis would be required to characterize the transcriptome in the transition or maturation stages of ameloblasts.

Enamel formation processes are coordinated by strictly synchronized functions of the dental epithelium (Krivanek et al., 2017). SI cells cover the ameloblast layer and may contribute to ameloblast differentiation through Shh signaling (Koyama et al., 2001). Our previous studies had demonstrated the role of SI in enamel mineralization by the regulation of alkaline phosphatase activity in a mouse model (Yoshizaki et al., 2014, 2017). Some reports had earlier indicated that SI cells regulate ion transporters associated with ameloblast (Liu H. et al., 2016). Solute carrier family member *Slc4a4* (NBCe1) is complementarily expressed in ameloblasts and SI cells and has implicated functions in pH regulation during enamel mineralization (Jalali et al., 2014), which is in agreement with our results (Figure 2F). Thus, the coordination of SI cells and ameloblasts is essential for enamel mineralization. In this study, we proposed *Cldn10* as a novel cell-type-specific gene of SI cells (Figure 3). CLDN10 mutation in humans causes Helix syndrome, which shows enamel wear as phenotype (Hadj-Rabia et al., 2018). Although the function of *Cldn10* during tooth development has not reported yet, *Cldn10* regulates ion permeability in kidney in mice (Breiderhoff et al., 2012). *Cldn10* may contribute to ion permeability in SI cells and may play essential roles in enamel formation.

The transcriptomes of SR and OEE cells are not as well characterized as those of other epithelial cell types; therefore, some part of their role during tooth development still remains hypothetical. The presence of large extracellular spaces may histologically identify SR cells. This space contains carbohydrates and glycocalyx-rich tissue fluid (Kallenbach, 1978). The glucose transporter 1, *Slc2a1* (GLUT1), is highly expressed in the early stage of SR cells (Supplementary Figure 5), and glucose uptake determines the size of tooth germ in *ex vivo* culture (Ida-Yonemochi et al., 2012). Some researchers had speculated SR cells to act as a carrier of nutrients to the enamel organ owing to the lack of blood supply in the latter (Ida-Yonemochi et al., 2005). In this study, GO terms in SI/SR cluster were enriched in nutrition-related functions (Figure 2F), thus reflecting the role of SR cells in supplying nutrition to the developing tooth germ.

Few reports had previously indicated the role of OEE in the regulation of tooth germ size. *Iroquois homeobox 1* (*Irx1*) transcription factor was expressed preferentially in OEE, SR, and SI cells (Supplementary Figure 5) and its disruption caused a delay of tooth growth in mouse (Yu et al., 2017). OEE cells are known to contribute to root formation by constituting Hertwig’s epithelial root sheath. During tooth development, cell proliferation markers such as Ki67 are strongly positive in IEE (Chiba et al., 2019); however, OEE proliferates more actively than IEE during the formation of Hertwig’s epithelial root sheath (Yokohama-Tamaki et al., 2006). Also, in pre-eruption molars, OEE seems to be potent for proliferation (Liu H. et al., 2016). GO terms of the IEE/OEE cluster were enriched in cell mitosis (Figure 2E). Collectively, OEE may have an important role in tooth size regulation. We further identified *Krt15* as an OEE-specific marker and *Atf3* as an SR/OEE marker (Figure 3A). Because the OEE-specific marker had not been established earlier, these findings contribute to uncovering the roles of OEE and SR cells. Krt15 is expressed in the progenitor cells of the skin, intestine, and esophagus (Giroux et al., 2017, 2018; Moon et al., 2019). Krt15 is localized in these epithelial basal layer and suggested to provide progenitor cells. Interestingly, Krt15-positive cells have resistant cell property for oncogenesis (Giroux et al., 2018; Moon et al., 2019). Given that OEE cells could provide progenitor cells (Liu H. et al., 2016), Krt15-positive cells may play a role in the maintenance of stemness, preventing oncogenesis in OEE.

Our transcriptome map reflected a new perspective of all stages of development of dental cells; however, we could not clearly distinguish between IEE, OEE, SI, and SR clusters (Figure 2C). The possible reason could be that we were able to capture only a limited number of these cell types in the incisor. Certain developmental stages of molars might show more clearly differentiated cell types, as well as clusters of each cell type, in scRNA-seq data.

Our scRNA-seq datasets clearly distinguished *Dspp* + ameloblast from Ambn + ameloblast (Figures 2C, 4A–C). This is the first report describing the novel subpopulation of ameloblasts, based on transcriptome analysis. The secretory stage of ameloblasts differentiated from pre-ameloblasts with an elongated shape. GO terms enriched in *Dspp* + early-differentiated ameloblasts might indicate the morphological change as “epithelial development” and “extracellular matrix organization” (Figure 5F). Interestingly, transport-related terms, such as “sodium ion transport,” “nephron epithelium development,” and “urogenital system development,” were highly enriched in *Dspp* + ameloblasts. This may imply the role of *Dspp* + ameloblasts in the structural reorganization of cytoplasm to prepare for the later stage of development. Ameloblasts secrete high amounts of enamel matrices and subsequently need to absorb the same into the cytosol (Bartlett, 2013). Therefore, *Dspp* + ameloblasts may act as a “preparative stage” for the establishment of a transport system. Further, *Dspp* + ameloblasts highly expressed *Shh* and *Wnt6a* (Figures 2D, 5E and Supplementary Figure 1). During tooth development, *Shh* and *Wnt6a* are expressed in enamel knot in molar and pre-ameloblast to ameloblast in incisor, and they promote ameloblast differentiation (Sarkar and Sharpe, 1999; Gritli-Linde et al., 2002; Wang et al., 2010). The *Dspp* + ameloblasts produce these growth factors and may promote further differentiation. Although P7 incisor does not form enamel knot, *Dspp* + ameloblasts may act as enamel knot-like cells to promote ameloblast differentiation for continuous incisor development.

In this study, we identified *Dspp* as the stage-specific marker gene of early-differentiated ameloblasts. *Dspp* mutation causes dentinogenesis imperfecta (DI) in humans (Lee et al., 2013), and the role of *Dspp* in dentin development is also well characterized (Sreenath et al., 2003). However, the role of *Dspp* in amelogenesis has not been reported yet. In the dental clinic, we often see patients with DI, having severe enamel fractures (Min et al., 2014). This may result from enamel abnormality in DI tooth. Because *Dspp* could play a potent role in enamel formation, further analysis of *Dspp* in amelogenesis might uncover a novel mechanism of the ameloblast development process. Therefore, our findings established the transcriptomic characteristics of dental epithelial cell types and provided a precious resource for further analysis of ectodermal organ development.

## Data Availability Statement

The datasets analyzed for this study can be found in the NCBI GEO: GSE146855.

## Ethics Statement

The animal study was reviewed and approved by the National Institute of Dental and Craniofacial Research (NIDCR) Animal Care and Use Committee.

## Author Contributions

YC: concept of experiments and writing the manuscript. KS and AY: writing manuscript. DM, EB, CR, KY, TN, and RM: experimental work. YY: concept of experiments. SF: concept of experiments, writing the manuscript, and supervision of study. All authors contributed to the article and approved the submitted version.

## Conflict of Interest

The authors declare that the research was conducted in the absence of any commercial or financial relationships that could be construed as a potential conflict of interest.

## Footnotes

1 http://software.10xgenomics.com/single-cell/overview/welcome

2 https://support.10xgenomics.com/single-cell-geneexpression/software/pipelines/latest/advanced/references

3 http://geneontology.org
