# Supplementary material for: Single-Cell RNA-Sequencing From Mouse Incisor Reveals Dental Epithelial Cell-Type Specific Genes
Source: Front Cell Dev Biol. 2020 Sep 1;8:841. doi: 10.3389/fcell.2020.00841 (PMC7490294; doi:10.3389/fcell.2020.00841)
Supplement: Supplementary file 1 [file Image_1.pdf]

Supplementary Material

Supplementary Figures

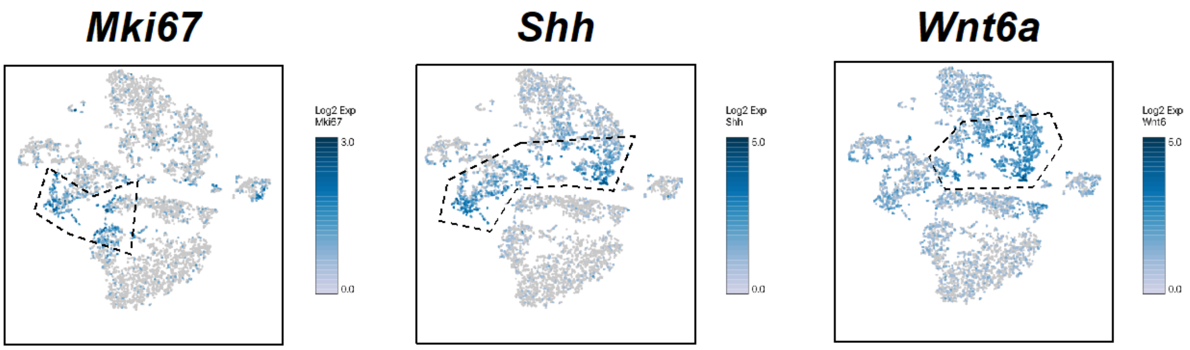

Supplementary Figure 1. Gene expression of *Mki67*, *Shh,* and *Wnt6a* in t-SNE plot. The expression of *Mki67*, *Shh,* and *Wnt6a* are shown. *Mki67* was highly expressed in IEE/OEE cluster. *Shh* was expressed in IEE/OEE and Ameloblast (I) clusters. *Wnt6a* was expressed in Ameloblast (I) cluster.

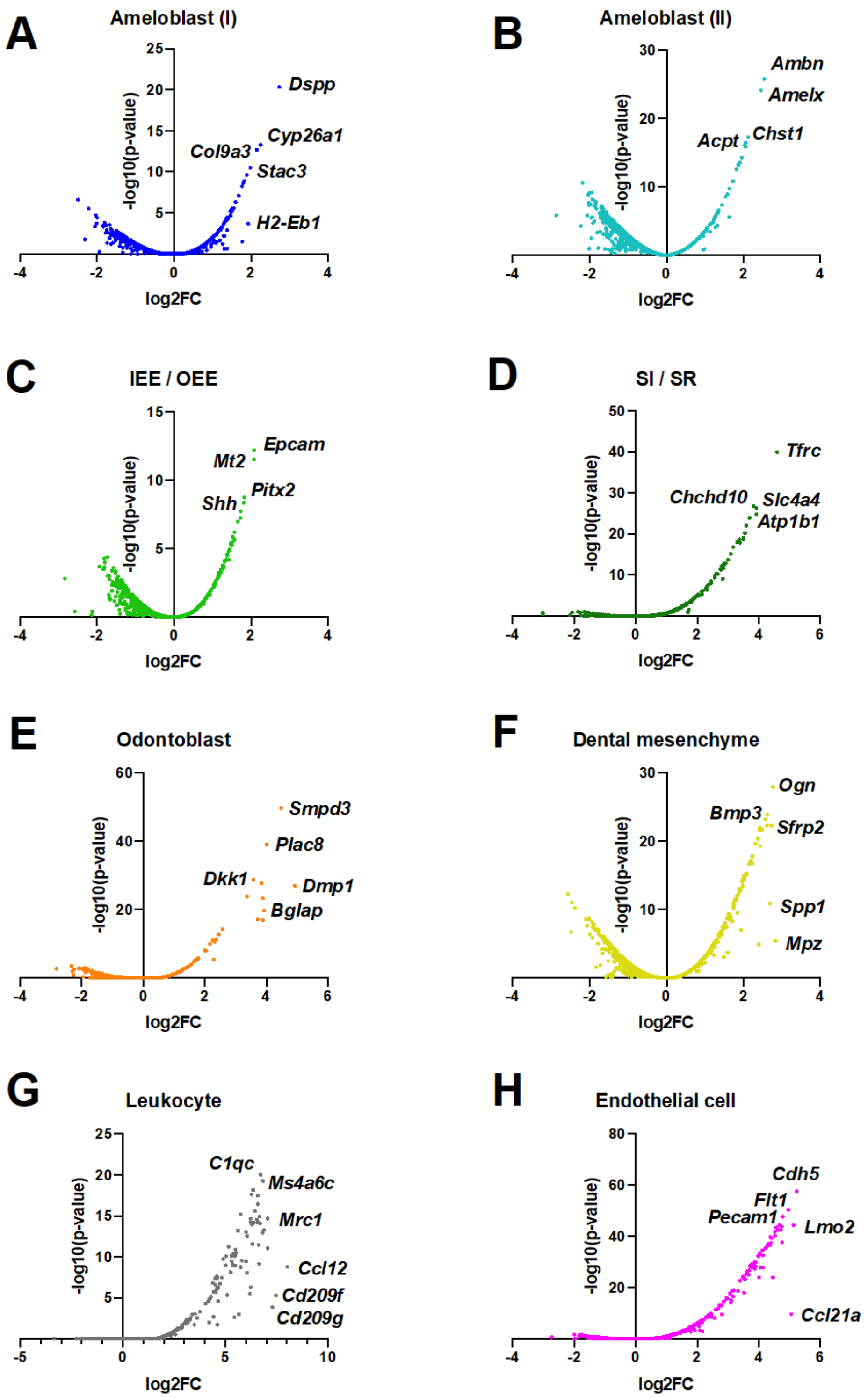

Supplementary Figure 2. All significantly differentially expressed genes from each clusters in volcano plot. Highly expressed genes are highlighted with gene name. (A) Ameloblast (I), (B) Ameloblast (II), (C) IEE/OEE, (D) SI/SR, (E) Odontoblast, (F) Dental mesenchyme, (G) Leukocyte, (H) Endothelial cells clusters.

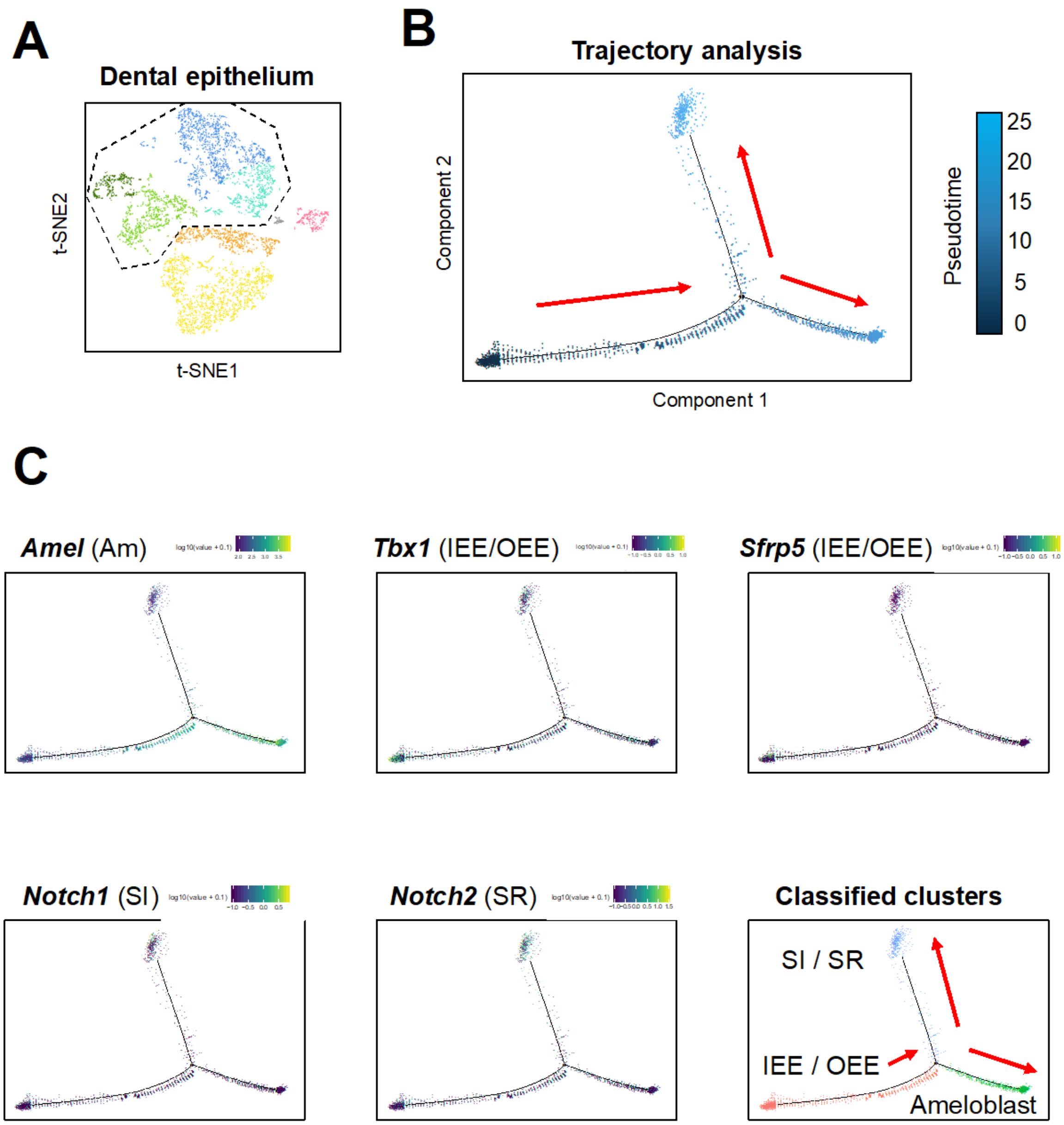

Supplementary Figure 3. Trajectory analysis of dental epithelial cells. (A) Dental epithelial cells were used for the trajectory analysis as input. Dotted line indicates input dataset. (B) Pseudo-temporal ordering of ameloblast lineages (n = 3,385) in trajectory plot. (C) Expression of dental cell-type specific marker genes projected onto the trajectory plot. Clusters are classified based on marker genes expression.

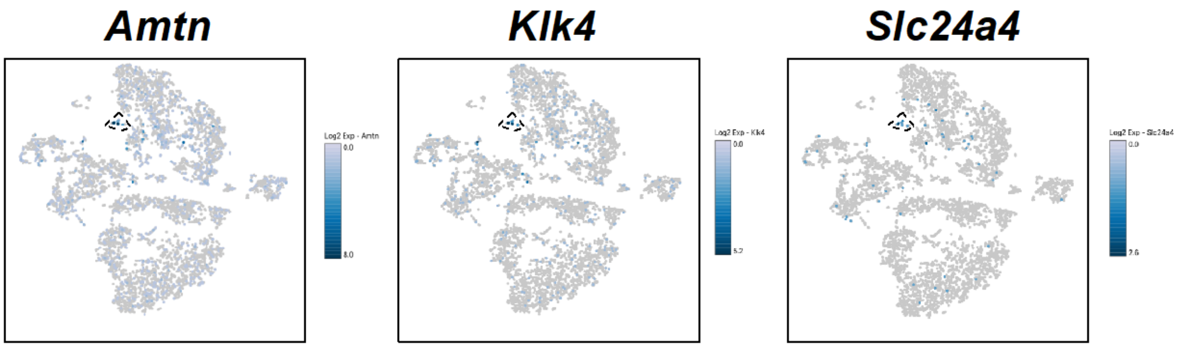

Supplementary Figure 4. Gene expression of transition and maturation stages of ameloblast in t-SNE plot. The transition stage of ameloblast marker, *Amtn* and *Klk4*, and maturation stage marker *Slc24a4* were expressed in a part of ameloblast cluster.

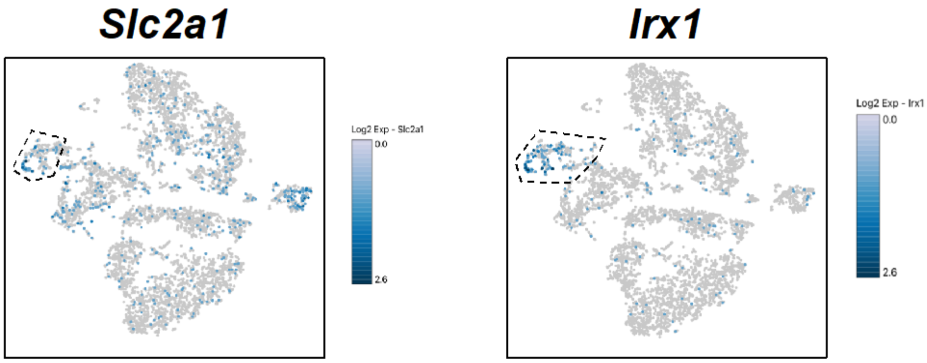

Supplementary Figure 5. Gene expression of *Slc2a1* and *Irx1* in t-SNE plot. The expression of *Slc2a1* and *Irx1* are shown. *Slc2a1* was highly expressed in SI/SR cluster. *Irx1* was expressed in SI/SR and OEE clusters.
